# Prevalence of COVID-19 fear and its association with quality of life and network structure among Chinese mental health professionals after ending China’s dynamic zero-COVID policy: a national survey

**DOI:** 10.3389/fpubh.2023.1280688

**Published:** 2023-10-30

**Authors:** Mei Ieng Lam, Pan Chen, Qinge Zhang, Sha Sha, Feng-Rong An, Zhaohui Su, Teris Cheung, Gabor S. Ungvari, Chee H. Ng, Yu-Tao Xiang, Yuan Feng

**Affiliations:** ^1^Unit of Psychiatry, Department of Public Health and Medicinal Administration, Institute of Translational Medicine, Faculty of Health Sciences, University of Macau, Macao, Macao SAR, China; ^2^Kiang Wu Nursing College of Macau, Macao, Macao SAR, China; ^3^Centre for Cognitive and Brain Sciences, University of Macau, Taipa, Macao SAR, China; ^4^Beijing Key Laboratory of Mental Disorders, National Clinical Research Center for Mental Disorders & National Center for Mental Disorders, Beijing Anding Hospital, Capital Medical University, Beijing, China; ^5^School of Public Health, Southeast University, Nanjing, China; ^6^School of Nursing, Hong Kong Polytechnic University, Kowloon, Hong Kong SAR, China; ^7^Section of Psychiatry, University of Notre Dame Australia, Fremantle, WA, Australia; ^8^Division of Psychiatry, School of Medicine, University of Western Australia, Perth, WA, Australia; ^9^Department of Psychiatry, The Melbourne Clinic and St Vincent's Hospital, University of Melbourne, Richmond, VIC, Australia

**Keywords:** fear, quality of life, COVID-19, mental health professionals, network analysis

## Abstract

**Background:**

China recorded a massive COVID-19 pandemic wave after ending its Dynamic Zero-COVID Policy on January 8, 2023. As a result, mental health professionals (MHPs) experienced negative mental health consequences, including an increased level of fear related to COVID-19. This study aimed to explore the prevalence and correlates of COVID-19 fear among MHPs following the end of the Policy, and its association with quality of life (QoL) from a network analysis perspective.

**Methods:**

A cross-sectional national study was conducted across China. The correlates of COVID-19 fear were examined using both univariate and multivariate analyses. An analysis of covariance (ANCOVA) was conducted to determine the relationship between fear of COVID-19 and QoL. Central symptoms were identified using network analysis through the “Expected Influence” of the network model while specific symptoms directly correlated with QoL were identified through the “flow function.”

**Results:**

A total of 10,647 Chinese MHPs were included. The overall prevalence of COVID-19 fear (FCV-19S total score ≥ 16) was 60.8% (95% CI = 59.9–61.8%). The binary logistic regression analysis found that MHPs with fear of COVID-19 were more likely to be married (OR = 1.198; *p* < 0.001) and having COVID-19 infection (OR = 1.235; *p* = 0.005) and quarantine experience (OR = 1.189; *p* < 0.001). Having better economic status (good vs. poor: OR = 0.479; *p* < 0.001; fair vs. poor: OR = 0.646; *p* < 0.001) and health status (good vs. poor: OR = 0.410; *p* < 0.001; fair vs. poor: OR = 0.617; *p* < 0.001) were significantly associated with a lower risk of COVID-19 fear. The ANCOVA showed that MHPs with fear of COVID-19 had lower QoL [*F* = 228.0, *p* < 0.001]. “Palpitation when thinking about COVID-19” was the most central symptom in the COVID-19 fear network model, while “Uncomfortable thinking about COVID-19” had the strongest negative association with QoL (average edge weight = −0.048).

**Conclusion:**

This study found a high prevalence of COVID-19 fear among Chinese MHPs following the end of China’s Dynamic Zero-COVID Policy. Developing effective prevention and intervention measures that target the central symptoms as well as symptoms correlated with QoL in our network structure would be important to address COVID-19 fear and improve QoL.

## 1. Introduction

The COVID-19 pandemic has been a major global public health challenge (1) which poses a substantial physical health threat and also results in significant mental health burden (2, 3). A unique characteristic of the pandemic (4) has been the widespread fear of COVID-19 in the community (5), which can be triggered by the lack of knowledge regarding the novel illness (6) and its potential life-threatening risk (7).

Notably, fear of COVID-19 has been more profound among healthcare professionals (HPs) compared to the general population (8), and ranked as the top mental health challenge among HPs (9). Healthcare professionals have reported moderate to high levels of COVID-19 fear during the pandemic (10–12), while still needing to provide healthcare to infected patients during this critical period, thus causing an increased vulnerability to negative psychological impacts (3) and work-related burden (13). In recent meta-analyses, fear of COVID-19 was associated with a broad range of mental health problems such as stress, anxiety, insomnia, and depression among both the general population and HPs (7, 14). Additionally, fear of COVID-19 was linked to a lower quality of life (QoL) among the general populations (15, 16) and higher levels of job stress (10, 17, 18), burnout (13, 17), and turnover intention (10, 19, 20).

A recent meta-analysis (12) reported that the highest level of COVID-19 fear was found in Asia compared to other continents. Further, the level of COVID-19 fear was significantly associated with the highest increase of infection in United States (21). As such, the massive and sudden COVID-19 surge that occurred after the end of China’s Dynamic Zero-COVID policy in China resulted in widespread fear of COVID-19. Since August 2021, China had effectively adopted the Dynamic Zero-COVID policy through strict quarantine and management measures to control COVID-19 transmission (22). As omicron variants were found to have less pathogenicity than original strains (23), the Chinese government formally ended the Dynamic Zero-COVID policy on January 8, 2023, and discontinued all centralized quarantine, contact tracing, and mass testing of nucleic acids (24). However, Omicron variants, due to its enhanced and faster transmission ability (25), rapidly resulted in a large-scale infection wave across China (26). Hence, a massive surge in infections occurred immediately within a short period, estimated to be between 167 and 279 million cases (27).

The mental health impact of the COVID-19 pandemic affected more people than the physical health impact (3). As the COVID-19 pandemic evolved, the demand for mental health services increased dramatically (28), and Mental Health Professionals (MPHs) played vital roles in the provision of mental health services (3). Apart from the excessive work burden, MPHs had to adapt to unfamiliar environments caused by changes in the delivery and settings of mental health services, as well as dealing with the fear of COVID-19 (29, 30). In response to the increasing mental health burden, MPHs and academic societies in China published various guidelines concerning the delivery of mental health services (i.e., outreach and hotline services) (5, 31). Although substantial measures to mitigate the psychiatric effects of the pandemic have been implemented, the psychological well-being of MHPs in hospitals and community centers has received little attention (30) compared to frontline HPs during pandemic (32). Moreover, to date, there is a lack of research on the impact of COVID-19 fear on QoL, and the relationship between COVID-19 fear and QoL among MHPs. Previous research only explored the impact of COVID-19 fear on health-related QoL among the general population (15, 16), work-related QoL among MPHs in Greece (13) and in nurses in Spanish (33). QoL refers to the perceived physical, material, social, and mental well-being over time by individuals (34, 35). Measuring QoL is critical for both assessing the population needs and informing policy decisions (36, 37). Thus, it is imperative to investigate the relevant correlates of COVID-19 fear among MHPs and its links with QoL after the end of China’s Dynamic Zero-COVID policy.

Network analysis (NA) provides a novel way to examine psychiatric problems conceptualized as causal interactions between symptom systems (38). In the network, the nodes represent the symptoms associated with a specific syndrome, and the edges represent the correlations between the symptoms (39), while the central node represents the most influential symptom in a network (40, 41) which can be prioritized for specific intervention based on the interconnection to other symptoms (42). NA has been widely utilized for various psychiatric disorders among multiple population subgroups during the COVID-19 pandemic (43–45). Previous network analyses focused only on the fear of COVID-19 symptoms network model among the general population in Iran, Bangladesh, and Norway (46), while a recent network analysis study examined the interrelationship between anxiety, depression, and QoL among HPs in China during the pandemic (47). However, to date, no studies have focused on the symptoms of COVID-19 fear or their links with QoL among MHPs in China following the end of China’s Dynamic Zero-COVID policy.

To address these gaps, this study (1) investigated the prevalence and correlates of COVID-19 fear among MHPs in China immediately after the end of China’s Dynamic Zero-COVID policy, (2) identified the most central symptoms of COVID-19 fear in the network model, and (3) analyzed the relationship between symptoms of COVID-19 fear and QoL.

## 2. Methods

### 2.1. Study design and participants

A cross-sectional, national survey was conducted by the panel members of the Psychiatry Branch of the Chinese Nursing Association and the Chinese Society of Psychiatry between January 22 and February 10, 2023 immediately after the end of the Dynamic Zero-COVID policy in China. To decrease the possibility of infection during the COVID-19 pandemic, as recommended in previous studies (44, 48, 49), a snowball convenience sampling method with the WeChat-based Questionnaire Star was adopted for this study. WeChat is one of the most popular communication methods and is widely used in clinical practice and continuing education in China (50, 51). During the COVID-19 pandemic, all health professionals in China were required to report their health status daily using WeChat. Hence, thus it could be assumed that all MHPs were WeChat users (48, 52). Questionnaire Star program is a commonly used research tool in China’s epidemiological survey (53). To be eligible, participants were: (1) adults aged 18 years or above; (2) MHPs (e.g., psychiatrists, nurses or technicians) who worked in psychiatric hospitals or in psychiatric departments of general hospitals in China during the COVID-19 pandemic; and (3) able to understand Chinese and provide written informed consent. The Ethics Committee of the Beijing Anding Hospital in China approved the study protocol and all participants provided electronic informed consent.

### 2.2. Measures

The sociodemographic data were collected, including age, sex, marital status, educational level, clinical work experience (years), living status, perceived economic and health status, previous COVID-19 infection, and experience of quarantine during the COVID-19 pandemic.

The fear of COVID-19 infection was assessed using the validated Chinese version of the Fear of COVID-19 Scale (FCV-19S) (4, 54) that has good reliability and validity among Chinese populations (55). The FCV-19S consisted of seven items, covering two dimensions: physical response (three items) of fear and thoughts of fear (four items) (4, 54). Each item was rated on a five-point Likert scale from 1 (“strongly disagree”) to 5 (“strongly agree”). The total score ranged from 7 to 35, with a higher score indicating greater fear of COVID-19 (4). A total score of FCV-19S of ≥16 was considered as “having COVID-19 fear,” which could significantly reflect the psychological impact of COVID-19 fear (55).

The assessment of the global Quality of Life (QoL) was based on the sum of scores for the first two items of the Chinese version of the World Health Organization Quality of Life-Brief Version (WHOQOL-BREF) (56–58). The Chinese version of the WHOQOL-BREFF has been validated in Chinese populations with good sensitivity and specificity (58, 59) with a higher total score indicating better QoL (56, 58).

### 2.3. Statistical analysis

#### 2.3.1. Univariate and multivariate analyses

Statistical analyses were conducted using SPSS version 22.0 for both univariate and multivariate analysis (SPSS Inc., Chicago, Illinois, United States). One-sample Kolmogorov–Smirnov tests were used to assess the distribution normality of continuous variables. The sociodemographic and clinical characteristics of participants with and without fear of COVID-19 infection were compared using independent sample t-tests or Mann–Whitney U tests for continuous variables and Chi-square tests for categorical variables. To determine the independent correlates of COVID-19 fear, a binary logistic regression analysis was conducted, using fear of COVID-19 infection as the dependent variable and variables with significant differences in univariate analyses as independent variables by applying an “Enter” method. The threshold for significant statistical differences was set at *p* < 0.05 (two-tailed).

#### 2.3.2. Network estimation

Network structure analysis was conducted using R software (version 4.2.2) (60). The fear of COVID-19 infection network structure was analyzed using a Graphical Gaussian Model (GGM) with graphic least absolute shrinkage and selection operator (LASSO) and an Extended Bayesian Information Criterion (EBIC) model (61), which could provide enhanced prediction accuracy, interpretability, and optimality of the network model (62). The network estimation was evaluated using the “estimateNetwork” function in the R package “bootnet” with “EBICglasso” as the default method (63). The visualization of the network was conducted using the R package “qgraph” (61) and optimized with the visual representation by “ggplot2” (61, 64). In a network model, each node represents an individual symptom of COVID-19 fear, while each edge represents the association between two symptoms. Thick edges indicate stronger correlations, while green edges indicate positive correlations and red edges indicate negative correlations (63). Expected Influence (EI) in the network model was used to determine central symptoms based on its reliability as an indicator of centrality (65); nodes with a greater EI were considered to be more important and influential (66). The value of predictability was indicated as the linkage between its neighboring nodes (66), which was calculated using the “mgm” package (67). Additionally, the ‘flow’ function in the R package “qgraph” was used to identify specific symptoms of COVID-19 fear that were directly associated with QoL (63).

To determine the stability and accuracy of the network model, the “bootnet” function in R package (Version 1.4.3) (61) was used with 1,000 permutations of the case dropping bootstrap procedure for each node. The stability of the network was assessed using a correlation stability coefficient (CS-coefficient). In the presence of a correlation greater than 0.7, a maximum proportion of cases could be dropped, indicating a 95% probability that the original centrality indices would be correlated with the centrality of subset networks (61). According to previous studies (43, 61, 66), a CS-coefficient value exceeding 0.25 was considered stable in the network model, while a value exceeding 0.5 was considered preferable. An edge accuracy estimate was derived using bootstrapped 95% confidence intervals (CIs), where a narrower CI would suggest a more trustworthy network (61). A non-parametric bootstrapped difference test was conducted to evaluate differences between edge pairs. The difference between two nodes or edges was significant if zero was excluded based on the 95% CI (61).

## 3. Results

### 3.1. Participant characteristics

A total of 11,524 MHPs were invited to participate in this study, of whom 10,647 met the study entry criteria and completed the assessment, with a participation rate of 98.0%. The demographic and clinical characteristics of the participants are shown in Table 1. In the study, the mean age of participants was 34.85 (SD = 8.395) years and 18.0% were males (*n* = 1,920). Most participants had at least a college degree (*n* = 10,809; 94.8%), were married (*n* = 7,722; 72.5%) and lived with others (*n* = 9,454; 88.8%).

**Table 1:** Demographic characteristics of the study sample.

Variables	Total (*N* = 10,647)		Without fear of COVID-19 infection (*N* = 4,170)		With fear of COVID-19 infection (*N* = 6,477)		Univariable analysis		
	*n*	%	*n*	%	*N*	%		*df*	*p*
Male	1,920	18.0	792	19	1,128	17.4	4.270	1	**0.039**
College and above	10,089	94.8	4,002	96	6,087	94	46.771	3	**<0.001**
Married	7,722	72.5	2,967	71.1	4,755	64.2	6.517	1	**0.011**
Living with others	9,454	88.8	3,704	88.2	5,750	88.8	12.895	3	**0.005**
Perceived economic status									
Poor	1,163	10.9	326	7.8	837	12.9	109.967	2	**<0.001**
Fair	8,826	82.9	3,499	83.9	5,327	82.2			
Good	658	6.2	345	8.3	313	4.8			
Perceived health status									
Poor	698	6.6	170	4.1	528	8.1	180.739	2	**<0.001**
Fair	7,559	71.0	2,819	67.6	4,740	73.2			
Good	2,390	22.4	1,181	28.3	1,209	18.7			
COVID-19 Vaccines injection	10,507	98.7	4,188	98.8	6,389	98.6	1.559	4	0.816
Having COVID-19 infection since 2019	9,858	92.6	3,817	91.5	6,041	93.4	11.113	1	**0.001**
At least 1-week quarantine experience during the COVID-19 pandemic	5,873	55.2	2,173	52.1	3,700	57.1	25.794	1	**<0.001**
	Mean	*SD*	Mean	*SD*	Mean	*SD*	*Z*	*df*	*p*
Age (years)	34.85	8.395	34.72	8.393	34.94	8.395	−1.389	-*	0.165
Work experience (years)	12.68	9.165	12.57	9.251	12.75	9.109	−1.569	-*	0.117
Global quality of life	6.15	1.589	6.54	1.697	5.90	1.462	−21.131	-*	**<0.001**

Bolded values: <0.05; df, Degree of freedom; SD, Standard deviation. ^*^Mann–Whitney U test.

### 3.2. Prevalence and correlates of having COVID-19 fear

The mean total score of FCV-19S was 17.36 ± 6.147 (95% CI = 17.25–17.48%) and the overall prevalence of COVID-19 fear (FCV-19S total score ≥ 16) was 60.8% (*n* = 6,477; CI = 59.9–61.8%). A summary of the differences between subgroups with and without fear of COVID-19 is provided in Table 1. Participants with fear of COVID-19 were more likely to be male (*p* = 0.039), married (*p* = 0.011), living with others (*p* = 0.005), and have college and above education level (*p* < 0.001), poorer perceived economic status (*p* < 0.001), poorer perceived health status (*p* < 0.001), COVID-19 infection (*p* = 0.001), at least 1-week quarantine experience during the COVID-19 pandemic (*p* < 0.001), and a lower mean QoL score (*p* < 0.001). After controlling for covariates (i.e., gender, education, marital status, living status, economic and health status, COVID-19 infection, and quarantine experience), the analysis of covariance (ANCOVA) showed that MPHs with COVID fear still had lower QoL score [*F* =228.0, *p* < 0.001].

Table 2 shows the results of the binary logistic regression analysis of the participants with fear of COVID-19 infection. Participants who were married (OR = 1.198; *p* < 0.001), had COVID-19 infection since 2019 (OR = 1.235; *p* = 0.005), had at least 1-week quarantine experience during the COVID-19 pandemic (OR = 1.189; *p* < 0.001) were significantly associated with a higher risk of COVID-19 fear. Additionally, participants with better economic status (e.g., good vs. poor: OR = 0.479; *p* < 0.001; fair vs. poor: OR = 0.646; *p* < 0.001) and health status (e.g., good vs. poor: OR = 0.410; *p* < 0.001; fair vs. poor: OR = 0.617; *p* < 0.001) were significantly associated with a lower risk of fear of COVID-19 infection.

**Table 2:** Independent correlates of COVID-19 fear among Chinese mental health professionals (*N* = 10,647).

Variables	Multivariate logistic regression analysis		
	*p*	*OR*	95% *CI*
Male	**0.004**	**0.859**	0.773–0.954
College and above	**<0.001**	**0.633**	0.524–0.765
Married	**<0.001**	**1.198**	1.083–1.326
Living with others	0.115	0.891	0.772–1.029
Perceived economic status	**-**	-	-
Poor	**-**	1.0	-
Fair	**<0.001**	**0.646**	0.561–0.744
Good	**<0.001**	**0.479**	0.387–0.592
Perceived health status	**-**	**-**	-
Poor	**-**	1.0	-
Fair	**<0.001**	**0.617**	0.513–0.742
Good	**<0.001**	**0.410**	0.337–0.50
Having COVID-19 infection since 2019	**0.005**	**1.236**	1.065–1.434
At least 1-week quarantine experience during the COVID-19 pandemic	**<0.001**	**1.189**	1.096–1.285

Bolded values: <0.05; CI, Confidence interval; OR, Odds ratio.

### 3.3. Network structure of symptoms of COVID-19 fear

**Figure 1:**
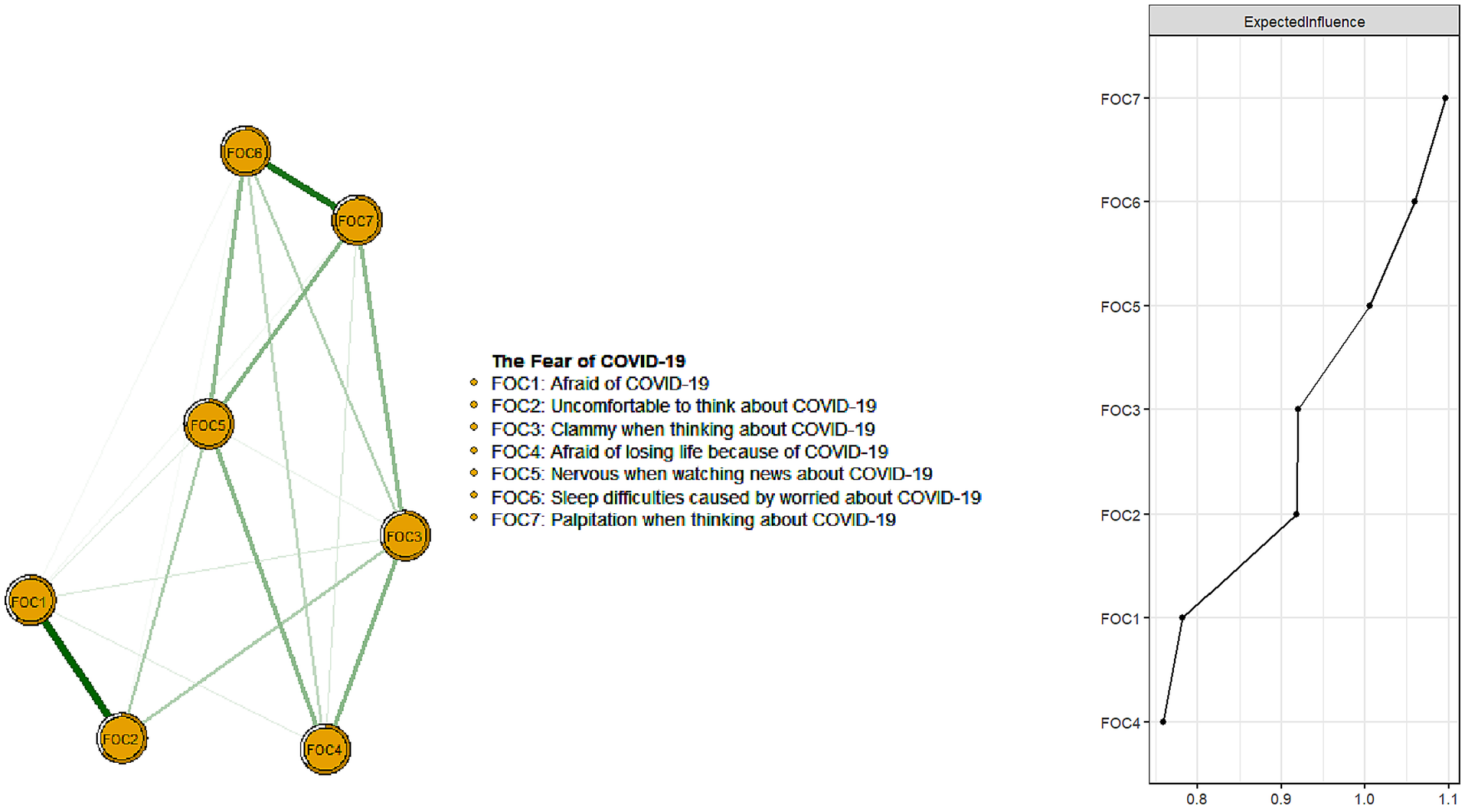
Network structure of the fear of COVID-19 infection among Chinese mental health professionals. Number of nodes: 8; Number of non-zero edges: 24/28; Mean weight/3ht: 0.1116963.

A network structure of fear of COVID-19 symptoms as measured by the FCV-19S items is shown in Figure 1. The mean predictability in this sample was 0.544, indicating that 54.4% of each node’s variance could be explained by its neighboring nodes. The three nodes that had the highest centrality measured by EI were FOC7 (“Palpitation when thinking about COVID-19”), FOC6 (“Sleep difficulties caused by worried about COVID-19”), and FOC5 (“Nervous when watching news about COVID-19”). Supplementary Table S1 shows descriptive information and network centrality indices for each symptom of COVID-19 fear, and Supplementary Table S2 presents the correlation matrix regarding the correlation coefficient between the seven items of the FCV-19S.

**Figure 2:**
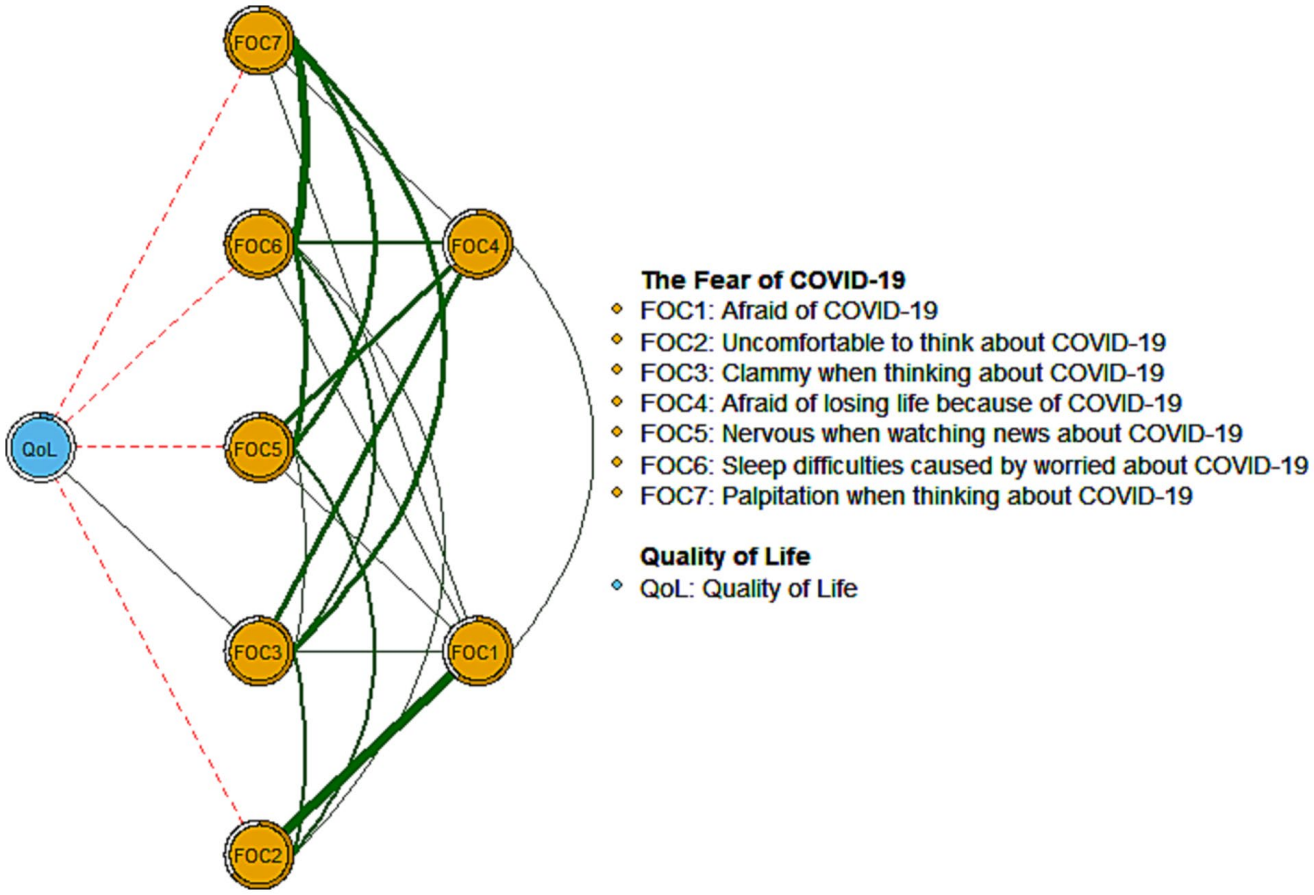
Flow network of quality of life and the fear of COVID-19 infection.

The flow network of QoL with symptoms of COVID-19 fear is presented in Figure 2. The FOC2 (“Uncomfortable to think about COVID-19”; average edge weight = −0.048) had the strongest negative association with QoL, followed by FOC7 (“Palpitation when thinking about COVID-19”; average edge weight = −0.043) and FOC5 (“Nervous when watching news about COVID-19”; average edge weight = −0.038).

**Figure 3:**
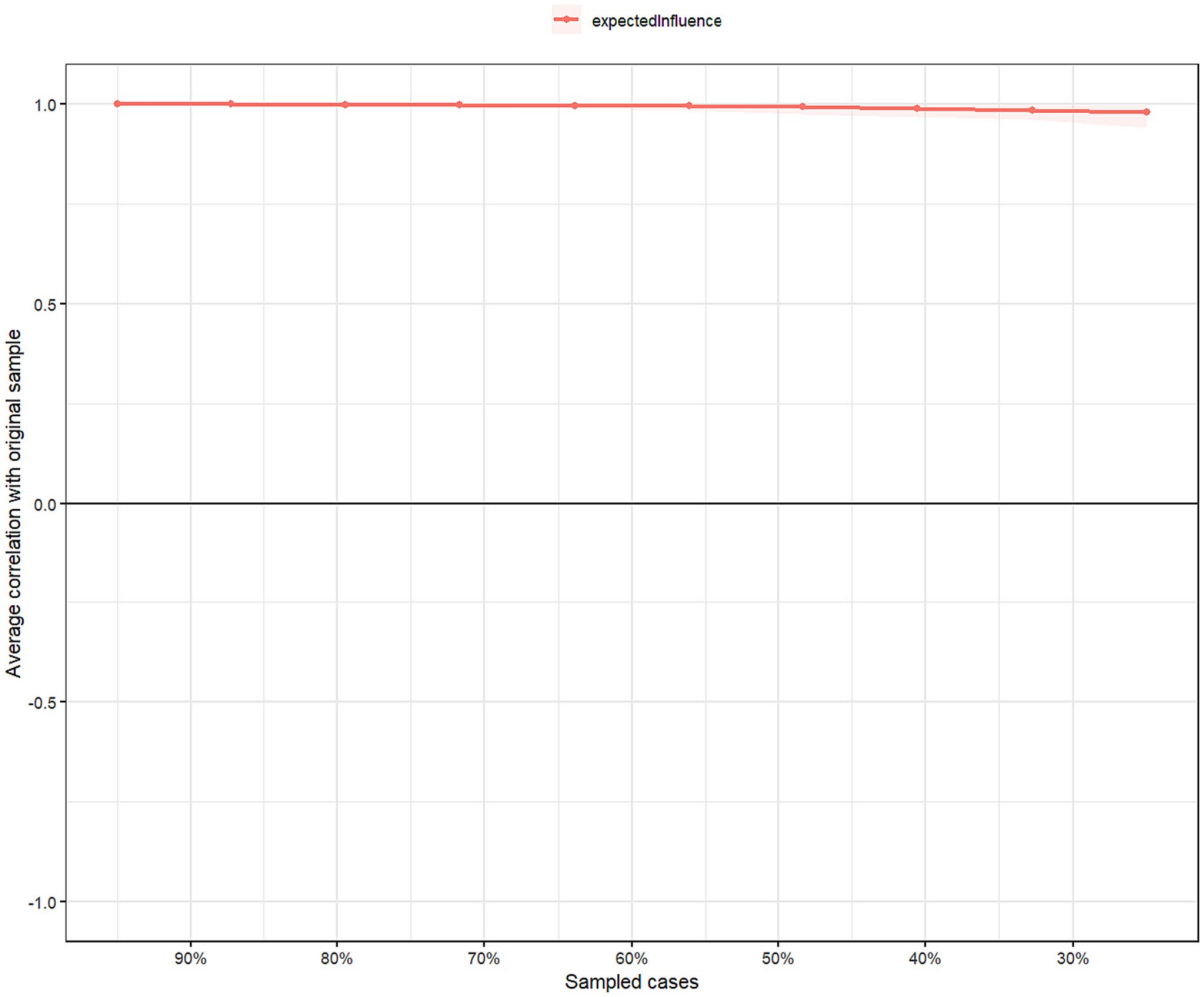
Network stability of COVID-19 fear among Chinese mental health professionals.

Figure 3 illustrates the result of network stability. The CS-coefficient of EI was 0.75 based on the case-dropping bootstrap procedure, indicating that the network model was stable even if 75% of the sample drooped without significantly affecting the network structure. For the network accuracy, bootstrap 95% CIs for estimated edge weights revealed a narrow range, as shown in Supplementary Figure S1. Most edge weights were non-zero, suggesting that the network was accurate and stable. Supplementary Figure S2 shows that most edge-weight comparisons were statistically significant using bootstrapped difference tests, indicating that the network model was reliable.

## 4. Discussion

This was the first study to explore the prevalence, correlates, and network structure of COVID-19 fear among MHPs. The prevalence of COVID-19 fear among MHPs was 60.8% (95% CI: 59.9–61.8%), which was similar to previous findings (63.2, 95% CI: 61–65.3%) among frontline nurses in Wuhan, China, during the Dynamic Zero-COVID policy in China (18). However, this figure exceeded those reported in studies of MHPs in Greece (23.7%; 95% CI:18.3–29.0%) (13), HPs in Bangladesh (27.3%; 95% CI:24.3–30.5%) (68), HPs and general population in Australia (31.9%; 95% CI:28.0–35.9%) (69), and also HPs and general population in India (54.8, 95% CI:52.3–57.3%) (70). The mean total score of FCV-19S was 17.36 ± 6.147 in this study (95% CI = 17.25–17.48%), which was higher than the mean total score of FCV-19S across 35 countries (13.11, 95% CI: 11.57–14.65%) among general and HPs population according to a recent meta-analysis (7). This finding indicates that there have been an escalation of COVID-19 fear among MHPs in China after the end of the Dynamic Zero-COVID policy. The high prevalence of COVID-19 fear may be explained by factors similar to those found in previous research among HPs, including the rapid and extensive spread of infection (4), increased work burden, inadequate Protective Equipment (PPE) and supports (71, 72), having conflicting information (73), and sense of stigmatization (73, 74). Taken together, these factors likely contributed to the increased fear of COVID-19 (75, 76). An appropriate fear response could decrease at-risk behavior or promote compliance with infection prevention strategies like social distancing and handwashing (77). However, an overreaction to COVID-19 fear would often lead to erratic behavior during infectious epidemics regardless of gender and social status (78), such as panic buying of household items (78, 79) and medical supplies (77). Measures that could reduce fear include having adequate infection control training, clear COVID-19 related protocols and precise communication to all employees (73, 78), priorities on work safety, support and a manageable workload, as well as peer support systems to assist with mental health issues (73).

The study also identified several correlates of COVID-19 fear among MHPs in China. We found that people who were married, had experienced COVID-19 infection or quarantine during COVID-19 pandemic were more likely to experience fear of COVID-19, which is consistent with previous findings (8, 11, 70, 80, 81). Reasons for their fear may include concerns about spreading the infection to their families (80, 81) and having increased sense of responsibility for their family during quarantine (80, 82). In addition, HPs who fear returning home due to the risk of infecting their family members have almost a twofold higher risk of experiencing psychological reactions, anxiety, and obsessive-compulsive symptoms (83). Participants with better economic and health status were less likely to fear COVID-19, which is aligned with previous research that linked lower income levels to higher rates of psychological distress among HPs (83), and found a higher level of fear among individuals with chronic diseases (16). The greater level of fear may be explained by the fact that individuals with low income were more vulnerable to having psychological distress (84), while individuals with physical comorbidities who were infected with COVID-19 were at higher risk of life-threatening complications (85).

The network structure of the fear of COVID-19 was also examined. “Palpitations when thinking about COVID-19” (FOC7) was the most central symptom of the fear of COVID-19 network model and had the strongest connection to other symptoms. Other influential nodes included “Sleep difficulties caused by worried about COVID-19” (FOC6) and “Nervous when watching news about COVID-19” (FOC5). These findings are aligned with previous network research on COVID-19 fear among the general population in Iran, Bangladesh, and Norway (46). The most common symptom of FCV-19S, palpitations, was observed in this study and also previous research among the general population (86) and HPs (87). Additionally, recent research showed that having palpitations was a significant predictor of anxiety, depression, and insomnia (88). They were more common in patients with anxiety or depression symptoms after COVID-19 infection due to COVID-19 fear (89).

“Sleep difficulty” refers to insomnia or hypersomnia (90). “Sleep difficulties caused by worries about COVID-19” (FOC6) was a central symptom, which supports the results of a recent meta-analysis that a high prevalence of insomnia (44.1, 95% CI: 31.3–57.0%) was observed among HPs during the COVID-19 pandemic (91). More than half of MHPs experienced insomnia in a United Kingdom study during COVID-19 pandemic (92). Sleep quality is an indicator of anxiety and depressive symptoms among HPs (93). Furthermore, palpitations and sleep difficulties are part of physical aspects of FCV-19S (4) as well as somatic symptoms (94). In particular, the prevalence rate of somatic symptoms among HPs was 16% (95% CI: 3–36%) during the COVID-19 pandemic in a previous meta-analysis (95), which may be triggered by the fear of COVID-19 (96). HPs were twice more likely to experience somatic symptoms if they were fearful of returning home due to the risk of infecting their family members (83). Somatic symptoms, sometimes referred to as somatization, are psychological defense mechanisms against the recognition or expression of psychological distress (i.e., fear) (97), particularly in cultures where psychiatric disorders are highly stigmatized (98, 99). Signs of somatization, such as palpation and sleep difficulty, may suggest that individuals have irrational fears associated with COVID-19 that should be addressed (46).

“Nervous when watching news about COVID-19” (FOC5) was another central symptom in our study. Certain news about COVID-19 may cause nervousness and pose a hindrance to reduce the fear of COVID-19, which may be related to the characteristics of the media. The relationship between HPs and media is complicated and can be both a source of support and stress (73). For example, the media has a role to advocate for healthcare workers, which could assist in mobilizing medical resources (i.e., PPE) (73). However, mass media also could enhance the fear of COVID-19, such as “coronavirus infodemic” defined as excessive reporting of inaccurate news over social media platforms which bred fear (100, 101). It could exacerbate psychological distress including anxiety phobia, panic, and depression (78, 102, 103). By portraying the news catastrophically during pandemics, the media perpetuated racism, stigma and xenophobia against particular communities (6). It was difficult for HPs to admit having psychological needs and to engage in psychological intervention due to the potential stigma exacerbated by the media representation of militarism among HPs (73). Thus, rather than focusing on specific treatment of COVID-19 fear, early screening on somatic symptoms (i.e., palpations and sleep difficulty), eradicating stigma around the mental health distress of MHPs and reducing the negative impact of fake news on social media as central symptoms may be more effective for prevention of COVID-19 fear.

There was a negative relationship between fear of COVID-19 and QoL, as observed in this study and also other studies (15, 16). In the flow network model, the fear of COVID-19 symptoms of “Uncomfortable to think about COVID-19” (FOC2), “Palpitation when thinking about COVID-19” (FOC7), and “Nervous when watching news about COVID-19” (FOC5) had the most robust, direct negative connection to QoL and would be potential targets for reducing fear and enhancing QoL in this population. Previous research indicated that psychological and emotional distress had a major effect on QoL among HPs during the COVID-19 pandemic (104). Dysfunctional worry (i.e., worry which affects QoL) corresponds to adverse emotional outcomes that might be detrimental to mental health (105). Additionally, somatic symptoms such as palpations are also key indicators of poor health-related QoL by mediating anxiety and depression (106). The three flow symptoms identified in this study are part of physical and psychological responses among FCV-19S (54), indicating that physiological and mental subconscious responses are probably triggered by fear arising from being confronted with possible harm associated with the specific threat (i.e., infectious diseases) (107). These fear responses are directly associated with an individual’s psychological adjustment skills (i.e., experimental avoidance and psychological resilience), which could be predicted by fear of COVID-19 (108). A previous study reported that HPs with avoidance experience had a higher risk of COVID-19 fear, whereas psychological resilience was a key protector for reducing these fears (108). Therefore, strengthening avoidance coping skills and psychological resilience might play an essential role in maintaining QoL (108, 109). For example, cognitive-behavioral therapy could significantly reduce experiential avoidance among patients with anxiety disorders (110), stress (111, 112), and depression among HPs, as well as result in a profound increase in psychological resilience among HPs (112). In tandem with those studies, intervention to reduce fear of COVID-19 among MPHs could provide an effective approach for enhancing the QoL of MPHs who suffer from excessive fear.

The strengths of this study included the large sample size and use of network analysis to identify central symptoms of COVID-19 fear and those with strong correlations with QoL. However, several limitations should be acknowledged. First, this study was cross-sectional, therefore, causal relationships between fear of COVID-19 and other factors could not be inferred. To analyze the causal relationships and dynamic changes in fear of COVID-19 over time, future longitudinal studies are necessary. Second, this study was conducted in China, so the results may not be representative of other regions due to differences in COVID-19 policies and trajectory. Third, this study did not collect data regarding rural and urban areas. Data on geographical areas and hospital types should be examined in future studies on COVID-19 fear among HPs. Fourth, snowball sampling via an online survey was used to reduce the risk of COVID-19 infection, which might have resulted in selection biases. Finally, the assessment based on self-report, might lead to recall bias and social desirability bias.

In conclusion, this study found a high prevalence of COVID-19 fear among Chinese MPHs immediately following the end of China’s Dynamic Zero-COVID policy, which was associated with poor quality of life. Being married, having COVID-19 infection, quarantine experience, lower economic and health status were observed to significantly increase the risk of COVID-19 fear. “Palpitation when thinking about COVID-19” (FOC7) was the most central symptom in the network model while “Uncomfortable to think about COVID-19” (FOC2) had the most robust correlation with poor QoL in this study. These symptoms might serve as possible targets in developing preventive strategies and treatments for MPHs with excessive fear of COVID-19. Future research should prioritize early screening of somatic symptoms, mental health stigma among MHPs and negative impact of social media to address the fear of COVID-19.

## Data availability statement

The datasets presented in this article are not readily available because The Ethics Committee of Beijing Anding Hospital in China that approved the study prohibits the authors from making publicly available the research dataset of clinical studies. Requests to access the datasets should be directed to xyutly@gmail.com.

## Ethics statement

The studies involving humans were approved by The Ethics Committee of the Beijing Anding Hospital in China. The studies were conducted in accordance with the local legislation and institutional requirements. The participants provided their written informed consent to participate in this study.

## Author contributions

ML: Data curation, Formal Analysis, Methodology, Writing – original draft. PC: Data curation, Methodology, Writing – review & editing. QZ: Methodology, Writing – review & editing. SS: Methodology, Writing – review & editing. F-RA: Methodology, Writing – review & editing. ZS: Data curation, Writing – review & editing. TC: Data curation, Writing – review & editing. GU: Data curation, Writing – review & editing. CN: Writing – review & editing. Y-TX: Methodology, Writing – original draft, Writing – review & editing. YF: Methodology, Writing – review & editing.

## Supplementary material

SUPPLEMENTARY FIGURE S1 Bootstrapped confidence intervals of edge weights.

SUPPLEMENTARY FIGURE S2 Estimation of edge weight difference by bootstrapped difference test.

SUPPLEMENTARY TABLE S1 Descriptive information and network centrality indices of COVID-19 fear.

SUPPLEMENTARY TABLE S2 Correlation matrix of the FCV-19S items.
