# Supplementary material for: Prevalence of COVID-19 fear and its association with quality of life and network structure among Chinese mental health professionals after ending China’s dynamic zero-COVID policy: a national survey
Source: Front Public Health. 2023 Oct 30;11:1280688. doi: 10.3389/fpubh.2023.1280688 (PMC10642929; doi:10.3389/fpubh.2023.1280688)
Supplement: SUPPLEMENTARY FIGURE S1 — Bootstrapped confidence intervals of edge weights. [file Table_1.docx]

**Supplementary materials**

Table S1. Descriptive information and network centrality indices of COVID-19 fear

Table S2. Correlation matrix of the FCV-19S items

Figure S1. Bootstrapped confidence intervals of edge weights

Figure S2. Estimation of edge weight difference by bootstrapped difference test

Table S1. Descriptive information and network centrality indices of the fear of COVID-19 infection

| Item | Item content | Mean (SD) | Prevalence ^a^ | Predictability | EI |
| --- | --- | --- | --- | --- | --- |
| FOC1 | Afraid of COVID-19 | 2.71 (1.058) | 85.0% | 0.631 | 0.782 |
| FOC2 | Uncomfortable to think about COVID-19 | 2.75 (1.079) | 84.8% | 0.592 | 0.918 |
| FOC3 | Clammy when think about COVID-19 | 2.34 (0.979) | 77.0% | 0.550 | 0.920 |
| FOC4 | Afraid of losing life because of COVID-19 | 2.37 (1.044) | 75.7% | 0.602 | 0.759 |
| FOC5 | Nervous when watching news about COVID-19 | 2.51 (1.050) | 79.7% | 0.516 | 1.006 |
| FOC6 | Sleep difficulties caused by worried about COVID-19 | 2.34 (0.994) | 76.7% | 0.461 | 1.060 |
| FOC7 | Palpitation when think about COVID-19 | 2.33 (0.995) | 76.4% | 0.456 | 1.096 |

Note:

^a^ The prevalence indicates the rate of the presence of each symptom with a score more than 0.

IQR: interquartile range; SD: standard deviation; EI: Expected influence.

**Table S2.** Correlation matrix of the FCV-19S items

|  | FOC1 | FOC2 | FOC3 | FOC4 | FOC5 | FOC6 | FOC7 |
| --- | --- | --- | --- | --- | --- | --- | --- |
| FOC1 | 0 | 0.561 | 0.071 | 0.054 | 0.057 | 0.018 | 0.022 |
| FOC2 | 0.561 | 0 | 0.168 | 0.000 | 0.170 | 0.014 | 0.000 |
| FOC3 | 0.071 | 0.168 | 0 | 0.254 | 0.037 | 0.156 | 0.237 |
| FOC4 | 0.054 | 0 | 0.254 | 0 | 0.246 | 0.133 | 0.072 |
| FOC5 | 0.057 | 0.170 | 0.037 | 0.246 | 0 | 0.233 | 0.258 |
| FOC6 | 0.018 | 0.014 | 0.156 | 0.133 | 0.233 | 0 | 0.502 |
| FOC7 | 0.022 | 0 | 0.237 | 0.072 | 0.258 | 0.502 | 0 |

Note:

FOC1: Afraid of COVID-19; FOC2: Uncomfortable to think about COVID-19; FOC3: Clammy when think about COVID-19; FOC4: Afraid of losing life because of COVID-19; FOC5: Nervous when watching news about COVID-19; FOC6: Sleep difficulties caused by worried about COVID-19; FOC7: Palpitation when think about COVID-19

Figure S1. Bootstrapped confidence intervals of edge weights


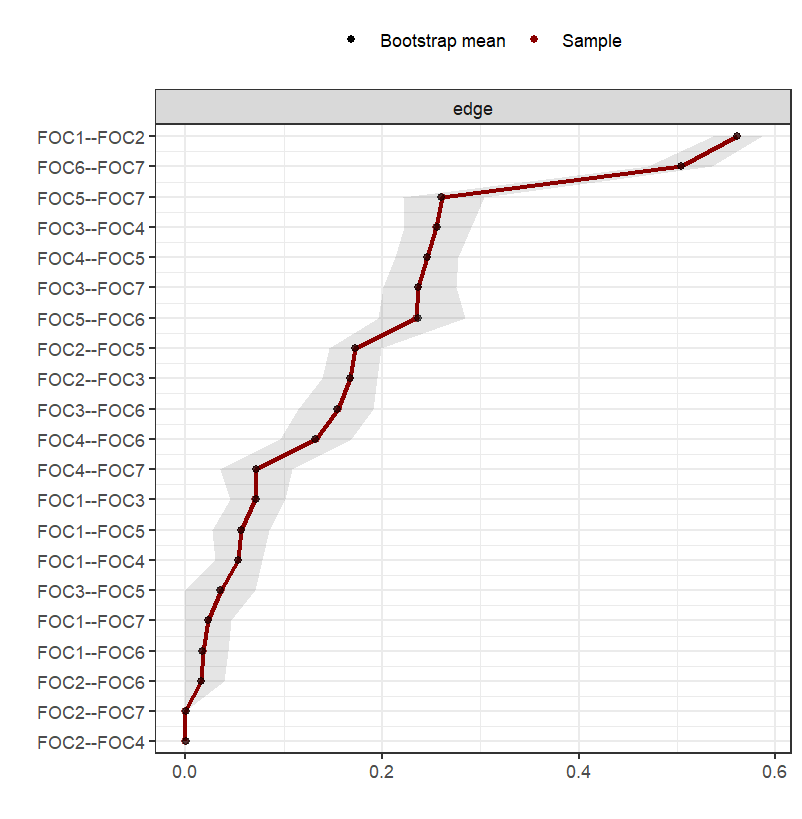


Notes: Each black dot represents the value of an edge weight, arranged from highest value to lowest value. With the non-parametric bootstrap procedure, the gray area represents 95% confidence intervals for edge weights. (FOC1: Afraid of COVID-19; FOC2: Uncomfortable to think about COVID-19; FOC3: Clammy when think about COVID-19; FOC4: Afraid of losing life because of COVID-19; FOC5: Nervous when watching news about COVID-19; FOC6: Sleep difficulties caused by worried about COVID-19; FOC7: Palpitation when think about COVID-19)

Figure S2. Estimation of edge weight difference by bootstrapped difference test


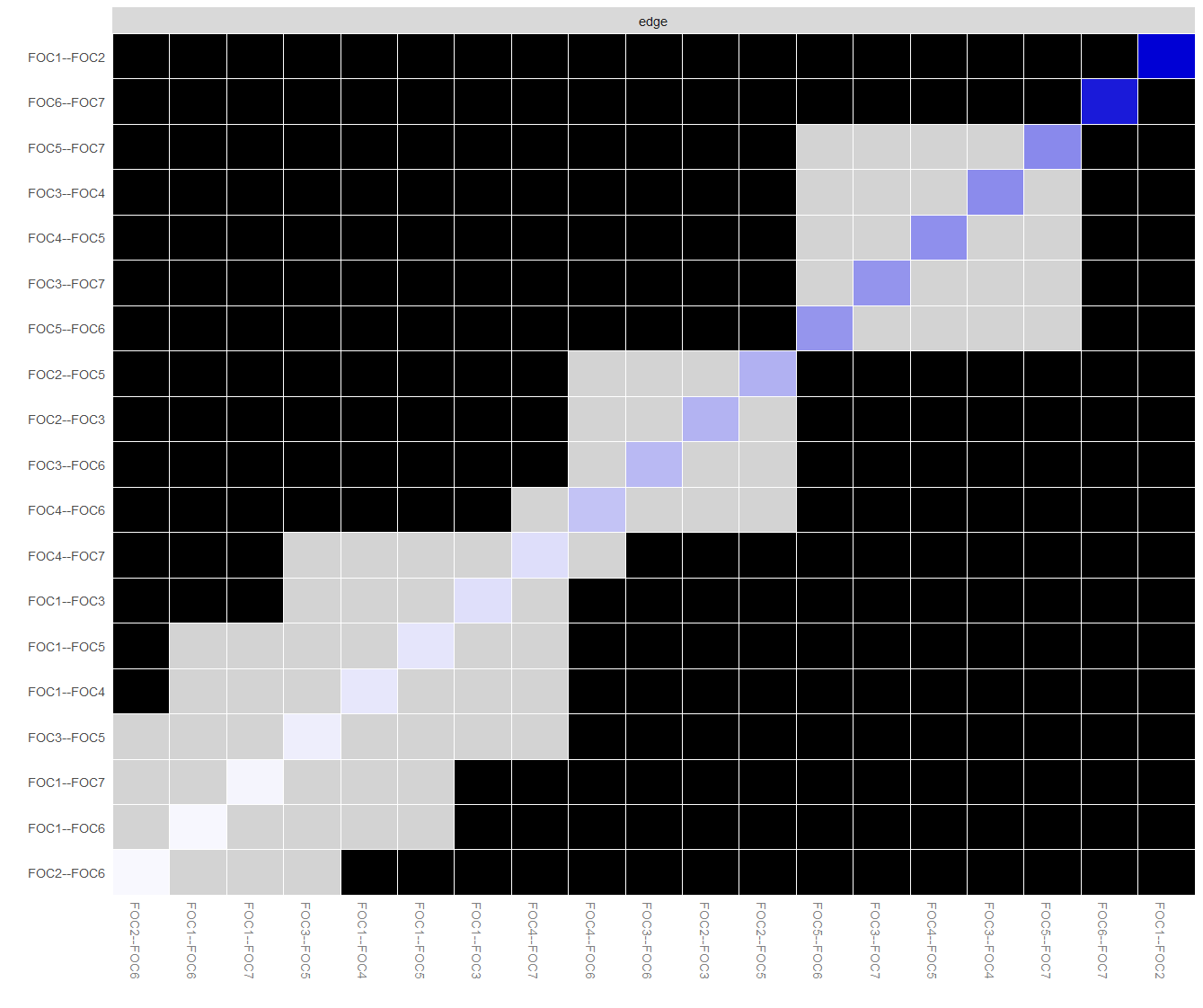


Notes: An edge with a gray box indicates that it does not significantly differ from another edge. The black boxes indicate edges that differ significantly from one another (α = 0.05). A blue box indicates a positive correlation in the edge-weight plot.
